# Identification of a novel immature dendritic cell subset with potential pro-leukemic effects in leukemia microenvironment

**DOI:** 10.1038/s41419-025-07851-2

**Published:** 2025-07-29

**Authors:** Xiaoxi Cui, Yifei Li, Wanzhen Xie, Ruiyun Li, Dongyue Zhang, Siqi Zhang, Qian Ren, Lina Wang, Guoguang Zheng

**Affiliations:** 1 State Key Laboratory of Experimental Hematology, National Clinical Research Center for Blood Diseases, Haihe Laboratory of Cell Ecosystem, Institute of Hematology & Blood Diseases Hospital, Chinese Academy of Medical Sciences & Peking Union Medical College, Tianjin, China; 2 Tianjin Institutes of Health Science, Tianjin, China

**Keywords:** Cancer microenvironment, Dendritic cells

## Abstract

Both intrinsic and microenvironmental factors contribute to the genesis and progression of leukemia. Dendritic cells (DCs) are important members of the immunomicroenvironment. The immature DCs (imDCs) and regulatory DCs (DCregs) participate in the formation of an immunosuppressive microenvironment that play adverse role in tumor progression. However, the characteristics of DCs in leukemia microenvironment have not been well established. Here, we identified a novel CD11c^+^MHCII^lo^ DC population (T-DC) accumulated in the mouse splenic T-ALL microenvironment. T-DCs exhibited an immature phenotype as they were characterized by low expression of MHCII molecules and co-stimulatory molecules such as CD86, CD83 and CD40. Database analysis revealed that low level expression of DC maturation-associated genes correlated with poor prognosis in leukemia patients. Furthermore, T-DCs promoted T-ALL progression contributed by their attenuated phagocytosis and CD4^+^ T cell activation potential. Moreover, RNA sequencing analysis demonstrated that T-DCs expressed low level of genes related to maturation and antigen processing. T-DCs showed similar expression pattern with DCregs and expressed high levels of immunosuppressive genes. In addition, single cell RNA sequencing demonstrated the heterogeneity of T-DCs, showing that they are mainly compose of cDC1s, cDC2s and macrophage-like DCs. Therefore, our findings uncover the critical role of a novel imDC subset in promoting leukemia progression through the suppression of T-cell immunity. These results may have significant implications for the development of immunotherapeutic strategies aiming at reversing immune evasion in leukemia and improving patient outcomes.

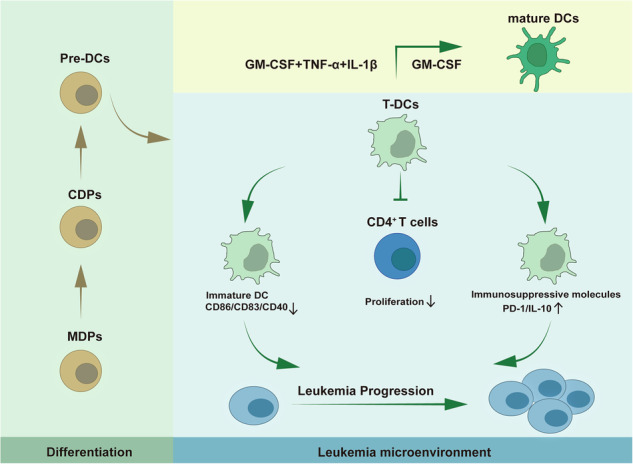

## Introduction

The occurrence and progression of diseases are greatly driven and impacted by both intrinsic and microenvironment factors. Different immune cells with heterogeneous functions orchestrate the specific immunomicroenvironments participating physiologic processes for the homeostatic maintenance as well as pathologic processes in diverse diseases including malignancies [1]. Among them, dendritic cells (DCs) are pivotal antigen-presenting cells that serve as a bridge between innate and adaptive immunity, playing critical roles in coordinating immune responses [2, 3]. DCs are broadly classified into two major subsets, the conventional DCs (cDCs) and the plasmacytoid DCs (pDCs), while the cDCs are further classified into cDC1 and cDC2 [4].

DCs take part in cancer immunoediting and play important pathologic roles in different phases of tumors. DCs exert their anti-tumor effects by effective antigen presentation and T cell activation. Upon immunosurveillance by DCs and other immune cells, tumor cells may be destroyed, which lead to the elimination of tumors [5]. However, during tumor progression, the tumor microenvironment (TME) is frequently reshaped to an immunosuppressive state favorable for the growth and metastasis of tumor cells [6]. Different mechanisms covering diverse immune cell types have been proposed. Among them, the TME impair the maturation of DCs through various mechanisms. The maturation process of DCs is a complex and highly regulated event that involves significant changes in morphology, phenotype, phagocytosis, secretory characteristics [7]. The immature DCs (imDCs) typically express low levels of maturation markers such as major histocompatibility complex (MHC) class II, CD80, CD86 and CD40. They are characterized by reduced antigen-presenting and phagocytic abilities leading to compromised immune responses [8]. The TME factors, such as hypoxia, nutrient deficiency, and accumulation of reactive oxygen species, inhibit the expression of co-stimulatory molecules. Moreover, imDCs also promote tumor progression by overexpressing Il-10, which suppresses T cell proliferation [9]. In addition, regulatory dendritic cells (DCregs), which possess immunosuppressive properties and facilitate immune evasion, have been identified [10, 11]. In fact, DCregs encompass a range of developmental stages, from immature, semi-mature to mature, suggesting potential overlaps with imDCs in terms of phenotype and function [12–15]. Therefore, restoring the anti-tumor effects of DCs in the TME could potentially improve the efficacy of cancer immunotherapies.

Compared with solid tumors, leukemia has unique pathologic microenvironment [16]. The immune cells in the leukemic microenvironment including DCs, macrophages, natural killer (NK) cells, and regulatory T cells (Tregs), exhibit heterogeneous characteristics and play pathologic roles during disease progression through different mechanisms [17–22]. As an important member of the leukemia microenvironment, bidirectional interactions between DCs and the microenvironment have been elucidated, *i.e*. the leukemia microenvironment recruits and educates DCs, which in turn reshape the leukemia microenvironment and contribute to disease progression. AML patients with FLT3-ITD had significantly higher frequencies of cDCs and pDCs than those without the mutation and the elevation was associated with a more aggressive disease phenotype and poorer prognosis [23]. Furthermore, more imDCs were detected in patients who experienced relapse [24, 25]. Moreover, DCs from thymic microenvironments in multiple murine T-ALL models directly supported the growth of T-ALL cells [26]. In addition, in vivo depletion of myeloid cells including DCs lead to a significant reduction in leukemia burden and prolonged survival in T-ALL mouse models [27]. These studies highlighted the significance of DCs in the leukemia microenvironment as well as their association with disease progression and relapse. However, a lot of fundamental phenomena and mechanisms, such as the phenotypic and functional characteristics of DCs in leukemia microenvironment, the leukemia-specific subpopulations, the transcriptomic heterogeneity and the interaction between DC subpopulations and other immune cells, have not been fully elucidated.

Here, we identified a novel CD11c^+^MHCII^lo^ DC population (T-DC), which exhibited an immature phenotype, in the mouse splenic T-ALL microenvironment. Database analysis revealed that low level expressions of DC maturation-associated genes correlated with poor prognosis in leukemia. Furthermore, T-DCs promoted T-ALL progression contributed by their attenuated phagocytosis and CD4^+^ T cell activation potential. Moreover, single cell RNA sequencing (scRNA-seq) demonstrated the heterogeneity of T-DC. Therefore, our study identifies a novel immature DC subset in the leukemia microenvironment, which provides important insights for the mechanisms of leukemia progression as well as immune-based approaches for leukemia therapy.

## Methods and materials

### Mice

C57BL/6J (CD45.2) and C57B6.SJL (CD45.1) mice were provided by the Animal Center of the Institute of Hematology and Blood Disease Hospital, CAMS & PUMC. OT-II mice were purchased from Cyagen Biosciences. 6-8 weeks old female mice were used and maintained in the specific pathogen-free animal facility. All procedures for the animal experiments involved in this study were approved by the Animal Care and Use Committee at the institute of Hematology & Blood Diseases Hospital.

### Cell culture

All cells were cultured in RPMI-1640 supplemented with 10% fetal bovine serum (FBS) and antibiotics in a humidified atmosphere of 5% CO_2_ at 37 °C. All culture supplies were endotoxin free.

### Mouse model

The Notch 1-induced T-ALL model was described previously [28]. Briefly, c-kit^+^ cells enriched from the Bone marrow (BM) of C57BL/6J mice were infected with retrovirus carrying intracellular domain of *Notch1* (ICN1), MSCV-ICN1-IRES-GFP, and transplanted into lethally irradiated C57BL/6J recipients, all of which would develop T-ALL. Then, GFP^+^ leukemia cells were sorted and transplanted into C57BL/6J or C57B6.SJL mice (1 × 10^6^ cells/mouse) without irradiation. The mice were sacrificed by carbon dioxide asphyxiation at the indicated time points. All procedures for animal experiments were approved by the Animal Care and Use Committees at the Institution.

### Isolation of CD11c^+^MHCII^lo^ cells

Mice were sacrificed and the spleen cells were harvested as single-cell suspensions in PBS with EDTA and filtered through graded nylon filters. After removal of red blood cells by ammonium chloride lysing buffer, cell suspension was subjected to FACS for analysis. For isolation of CD11c^+^MHCII^lo^ cells, CD11c^+^ cells were first enriched with microbeads (Miltenyi Biotec, Bergisch Gladbach, Germany) and CD11c^+^MHCII^lo^ cells were finally sorted by FACS. We abbreviated CD11c^+^MHCII^lo^ cells, mature cDCs sorted from healthy and leukemia mice as T-DCs, N-mDCs and T-mDCs, respectively (day 15).

### Flow cytometric analysis, cell sorting

The BD FACS Canto II, LSR II and Fortessa flow cytometers (BD Biosciences, San Jose, CA) were used for FACS analysis. The FACS Aria III (BD Biosciences, San Jose, CA) was used for cell sorting. Standard protocols were followed for both FACS analysis and sorting. Flowjo software (version 10.8.1) was used for data analysis. The antibody information is provided in the Supplementary Table S1.

### Phagocytosis assay

Latex bead uptake experiments were used to measure the phagocytosis of DCs. DCs sorted from the spleens of normal and leukemic mice on day 15 were incubated with FITC-labeled 2-μm latex beads (Sigma-Aldrich) for 20 min at 37 °C before FACS analysis.

### T-cell proliferation assay

The experiments were performed as previously described [29]. The naïve T cells were obtained from C57BL/6J mice in DC and autologous T cell co-culture assays or from BALB/C mice in allogeneic mixed lymphocyte reaction (MLR) assays. Then, 1 × 10^5^ CFSE (2.5 μM; Thermo Fisher Scientific)-labeled CD4^+^CD44^-^CD62L^+^ naïve T cells were co-cultured with 5 × 10^4^ N-mDCs, T-DCs and T-mDCs in a 96-well plate in the presence of (4 × 10^5^ Beads/mL, 4.5μm Dynabeads Mouse T-Activator CD3/28, GIBCO) and T-cell survival factors IL-2 and IL-7 (100 ng/ml, Pepro Tech, Rocky Hill, NJ, USA) for 5 days, respectively. T cell proliferation and activation were measured by FACS as the CFSE fluorescence intensity attenuation. In in vitro DC-mediated antigen-specific T-cell proliferation assays, 5 × 10^4^ N-mDCs, T-DCs and T-mDCs were loaded with OVA_323-339_ (100 μg/ml, Sigma-Aldrich) overnight before coculture. Then 1 × 10^5^ Far Red (1 μM, Thermo Fisher Scientific)-labeled CD4^+^CD44^-^CD62L^+^ naïve T cells from OT-II mice were co-cultured with N-mDCs, T-DCs and T-mDCs, respectively in a 96-well plate for 5 days. DC-mediated antigen-specific naïve T-cell proliferation was measured by FACS as the Far Red fluorescence intensity attenuation.

### Identification of the precursors of T-DCs

For in vivo differentiation analysis, 1 × 10^4^ monocyte and DC progenitor cells (MDPs), common dendritic cell progenitors (CDPs), dendritic cell precursors (Pre-DCs), monocytes, cDCs and pDCs were sorted from CD45.2^+^ mice and intravenously injected into CD45.1^+^ recipients suffering T-ALL (day 11). Spleen cells of the recipients were collected on day 15 and the existence of CD45.2^+^ cells in the T-DCs population were analyzed by FACS.

### In vivo functional analysis of T-DCs

1 × 10^4^ T-ALL cells were intravenously injected alone or with 1 × 10^4^ T-DCs into recipient mice. PB GFP^+^ cell levels were monitored on days 14, 18 and 22. Mice were sacrificed and GFP^+^ cell levels in BM and spleen were analyzed on day 20. Tissues were collected and standard pathologic analysis was performed.

### cDNA synthesis and quantitative reverse-transcription PCR

Total RNA was isolated using RNeasy Mini Kit (Qiagen). cDNA was synthesized using Transcript All-in-one First-Strand cDNA Synthesis SuperMix (TransGen Biotech, China) following the manufacturer’s protocols. SYBR Green Kit (TaKaRa, China) was used for qRT-PCR experiments, which were performed on QuantStudio 5 (Thermo Fisher Scientific, USA). The expression levels of target genes were analyzed by the relative quantity (RQ) value calculated using the _ΔΔ_Ct method [_ΔΔ_Ct= (Ct_TARGET_-Ct_GAPDH_) _sample_- (Ct_TARGET_-Ct_GAPDH_) _calibrator_]. The sequences for all primers are shown in Supplementary Table S2.

### RNA sequencing (RNA-seq) and data analysis

RNA-seq was carried out by the Novogene CO., Ltd. (Beijing, China) following standard protocols. The RNA-seq data are available in the National Center for Biotechnology Information Gene Expression Omnibus database under accession number GSE285239.

### Single cell RNA sequencing

T-DCs were sorted from leukemia mice. Cell viability was examined by an TC10 automated cell counter (Bio-Rad). The cell suspension was loaded into Chromium microfluidic chips with v3 chemistry and barcoded with a 10× Chromium Controller (10× Genomics). RNA from the barcoded cells was subsequently reverse-transcribed and sequencing libraries were constructed with reagents from a Chromium Single Cell 3’ v3 reagent kit (10× Genomics) according to the manufacturer’s instructions. Sequencing was performed using Illumina HiSeq 2000 according to the manufacturer’s instructions. The scRNA-seq data reported in this paper have been deposited in the OMIX, China National Center for Bioinformation/Beijing Institute of Genomics, Chinese Academy of Sciences (https://ngdc.cncb.ac.cn/omix: accession no. OMIX008392).

### scRNA-seq-based data analysis

The scRNA-seq data was analyzed as previously described [30]. The quality of raw reads was detected with FastQC, and low-quality reads, adapter sequences, reads that below the 26 bases long and could not form pairs were removed through Trimmomatic software. Cell Ranger software (version 3.1.0, 10× Genomics) was used to perform alignment, filtering, barcode counting and UMI counting. Chromium cellular barcodes were used to generate feature barcode matrices, determine clusters, and perform gene expression analysis. Secondary analysis of gene expression was performed with the filtered gene-barcode matrix using the Seurat [31]. Genes with expression in fewer than 3 cells and cells that had less than 200 expressed genes were excluded. For clustering, highly variable genes were selected and the principal components based on those genes used to build a graph, which was segmented with a resolution of 0.6. Gene Ontology (GO) enrichment analysis of marker genes was implemented by the R package. GO terms with corrected *P* value less than 0.05 and the log fold change more than 0.25 were considered significantly enriched by marker gene. The *t*-distributed stochastic neighbor embedding (t-SNE) was used to visualize the clusters. The scaled expression levels of markers across different clusters were displayed in a heatmap. The connectivity map (CMap) analysis was conducted to explore the subgroup of T-DCs using R programming. The differentially expressed genes (DEGs) among T-DC Cluster 1–6 subpopulations were identified using the following selection criteria: *P*-value less than 0.05 for statistical significance and log fold change more than 0.25 to ensure biologically relevant expression differences. Connectivity score was performed as described by Schlitzer et al. [32], demonstrating the association between 6 subpopulations and the corresponding gene set, which included cDC1, cDC2, macrophages, and other related immune cell types [33–38] (Supplementary Table S4). Connectivity scores were plotted to compare lineage-specific enrichment across different subsets using ggplot2 (version 3.5.1).

### Statistical analysis

Results are shown as means ± SEM. GraphPad Prism 9.4.1 (GraphPad Software, CA) was used for data analysis. An unpaired Student’s *t* test (for two-group comparisons) or a one-way ANOVA (for more than two-group comparisons) was performed by GraphPad Prism to calculate the statistical significance. Survival time was compared by Kaplan–Meier analysis. *p* < 0.05 were considered significant. All experiments were repeated two to three times. All analyses were performed in R (version 4.4.2) with reproducible workflows.

## Results

### Identification of a new cDC population in T-ALL microenvironment

**Fig. 1:**
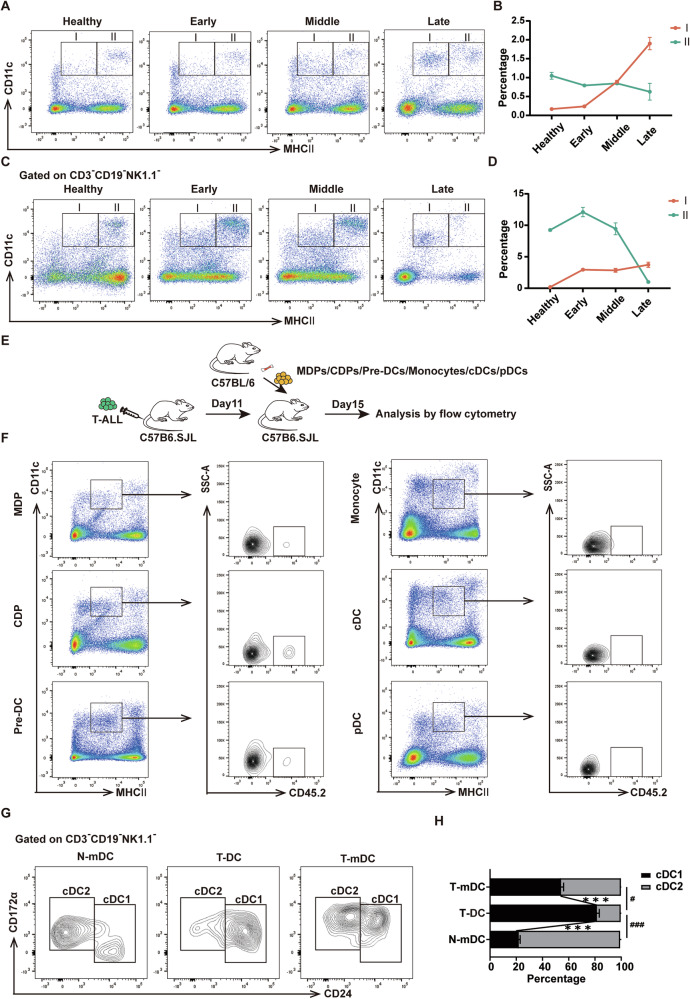
Identification of a new cDC population in T-ALL microenvironment. T-ALL mice were sacrificed at indicated stages of leukemia and mouse spleen samples were analyzed by flow cytometry analysis in the absence (**A**, **B**) or in the presence (**C**, **D**) of CD3, CD19 and NK1.1 antibodies. The representative flow cytometry results (**A**, **C**) show the CD11c^+^MHCII^lo^ (I) and CD11c^+^MHCII^+^ (II) populations and the percentages of them (**B**, **D**) are plotted. (*n* = 3) **E**, **F** 1 × 10^4^ MDPs, CDPs, Pre-DCs, monocytes, cDCs or pDCs from C57BL/6 mice (CD45.2) were transplanted into C57B6.SJL mice (CD45.1) on day 11 after transplantation of T-ALL cells. The spleen samples were analyzed by flow cytometry on day 15 (*n* = 3). The experimental design (**E**) is shown and the representative flow cytometry results (**F**) indicate the presence of CD45.2^+^ cells in the CD11c^+^MHCII^lo^ population. The representative flow cytometry results (**G**) and the percentages (**H**) of cDC1 and cDC2 in N-mDCs, T-DC and T-mDCs are shown. The data are representative of three independent experiments. *p < 0.05, ***p < *0.001*.

To study the role of DCs during the progression of T-ALL, the non-irradiated mouse T-ALL model was established as previously described (Supplementary Fig. S1A) [28]. The percentage of GFP^+^ leukemia cells in peripheral blood (PB) and spleen was detected to monitor leukemia progression (Supplementary Fig. S1B, C). Based on the level of leukemia cells in spleen, days 7, 11 and 15 were suggested as typical time points for early, middle and late stages of leukemia, respectively. Splenomegaly was observed, especially at late stage (Supplementary Fig. S1D). cDCs in the leukemia microenvironment were studied by using typical markers, MHCII and CD11c. Interestingly, besides the existence of CD11c^+^MHCII^+^ cDCs, accumulation of CD11c^+^MHCII^lo^ population was detected during leukemia progression (Fig. 1A, B, Supplementary Fig. S1E). Furthermore, this population were also detected when T, B and NK cells were excluded by using markers of CD3, CD19 and NK1.1 (Fig. 1C, D). Due to their expression of CD11c, these cells should be myeloid cells, and might be a cDC subpopulation. Therefore, different precursors sorted from CD45.2 mice, including MDPs, CDPs, Pre-DCs, monocytes, cDCs and pDCs, were intravenously transplanted into CD45.1 mice at the middle stage of leukemia (Fig. 1E, Supplementary Fig. S1F, G). Four days later, CD45.2 cells were analyzed in the CD11c^+^MHCII^lo^ population. The results showed that CD45.2 cells were detected in the MDP, CDP and Pre-DC groups but not in the monocyte, cDC or pDC group (Fig. 1F). These results indicate that this population can differentiate from DC precursors, suggesting that they should be a cDC subpopulation. We abbreviated this population, mature cDCs from healthy and leukemia mice as T-DCs, N-mDCs and T-mDCs, respectively. To further classify this population, cDC1 and cDC2 were analyzed in the above three populations. T-DCs consisted of both cDC1 and cDC2, but the majority was cDC1, which was significantly different from N-mDCs or T-mDCs (Fig. 1G, H). Furthermore, T-DCs did not express Siglec H, a marker of pDC (Supplementary Fig. S1H). Collectively, these results demonstrate that the specific population from T-ALL microenvironment is consistent with the characteristics of cDCs in terms of origin, markers and subpopulation distribution.

### T-DCs exhibit an immature phenotype

**Fig. 2:**
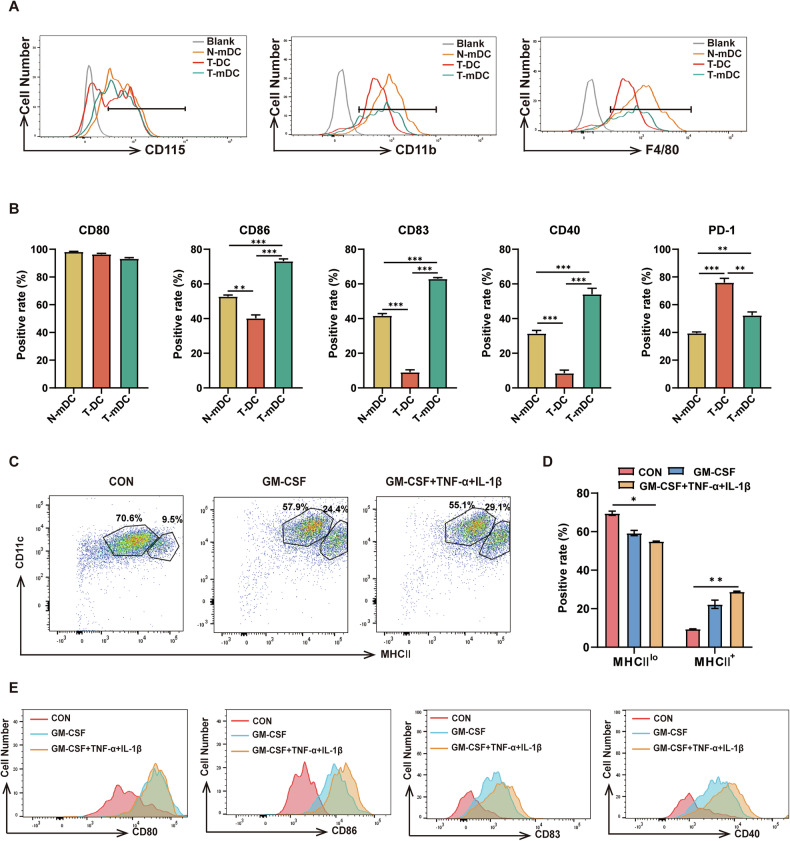
T-DCs exhibit an immature phenotype. **A** The expressions of CD115 (left), CD11b (medium) and F4/80 (right) in N-mDCs, T-DC and T-mDCs were assessed by flow cytometry. **B** The expressions of CD80, CD86, CD83, CD40 and PD-1 in N-mDCs, T-DC and T-mDCs were assessed by flow cytometry and the positive rates are plotted. **C**–**E** T-DCs were isolated and cultured in the absence (CON) or in the presence of maturation inducer (GM-CSF, GM-CSF + TNF-α + IL-1β) for 3 days. The representative flow cytometry results (**C**) are shown and the positive rates (**D**) of MHCII^lo^ and MHCII^+^ cell subsets are plotted. The expressions of CD80, CD86, CD83 and CD40 in MHCII^+^ cell subsets after induction were analyzed by flow cytometry (**E**). The data are representative of three independent experiments. *p < *0.05*, **p < 0.01, ***p < *0.001*.

To further investigate the phenotype of T-DCs, the expressions of CD115, CD11b and F4/80, which were expressed on the surface of myeloid cells, were examined. T-DCs expressed lower levels of CD115, CD11b and F4/80 than N-mDCs or T-mDCs (Fig. 2A). Furthermore, the expressions of CD80, CD86, CD83 and CD40, which were the antigen presenting cell (APC) specific co-stimulatory molecules relevant to the activation and maturation of DCs, were also detected. T-DCs expressed lower levels of CD86, CD83 and CD40 than T-mDCs (Fig. 2B). Moreover, T-DCs expressed higher level of PD-1, an immunosuppressive marker, than N-mDCs or T-mDCs (Fig. 2B). In addition, T-DCs expressed lower level of MHCII, an important marker of mature DCs, than N-mDCs or T-mDCs (Fig. 1A–D). Finally, sorted T-DCs were cultured with either GM-CSF alone or GM-CSF plus TNF-α and IL-1β, the typical DC maturation inducers, in the in vitro induction experiments. The increased proportion of MHC II^+^ cells as well as the up-regulated expressions of CD80, CD86, CD83 and CD40 were detected (Fig. 2C–E). These results suggest that T-DCs exert an immature phenotype.

### ALL patients with lower level of DC maturation-associated genes have worse prognosis

**Fig. 3:**
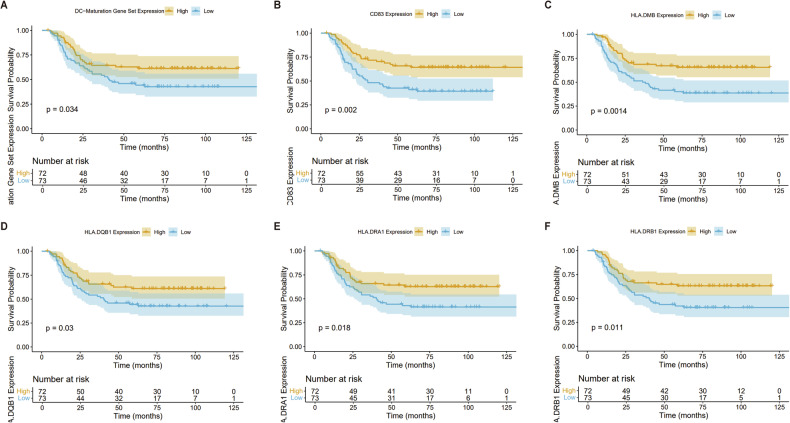
Low expression of mature DC-related genes correlates with poor prognosis in human ALL. The data of ALL patients from TARGET datasets (https://ocg.cancer.gov/programs/target) were analyzed (*n* = 145). **A** The survival of ALL patients is shown based on the expressions of genes within DC maturation-associated gene set. The survival of ALL patients is shown based on their expression of CD83 (**B**), HLA-DMB (**C**), HLA-DQB1 (**D**), HLA-DRA (**E**), and HLA-DRB1 (**F**) by Kaplan–Meier analysis.

To explore the clinical significance of immature DC in human ALL, a DC maturation-associated gene set, including CCL19, CCL21, CCR7, CD209, CD40, CD80, CD83, CD86, HLA-DMB, HLA-DRA1, HLA-DRB1, HLA-DQB1 and RELB, was defined based on the reports [39–43] (Supplementary Table S3). The expression levels of these genes and survival data were extracted from the open clinical database TARGET. Survival analysis revealed that ALL patients in the low score group had a worse prognosis than the high score group (*p* < 0.05) (Fig. 3A). To further delineate the contribution of individual gene within the maturation gene set, separate analyses were performed. ALL patients in the low score group of CD83, HLA-DMB, HLA-DQB1, HLA-DRA1 or HLA-DRB1 had a worse prognosis than the respective high score group (Fig. 3B–F). It’s worth noting that these genes are involved in antigen presentation and immune activation, suggesting the potential role of immature DC on the progression of ALL.

### T-DCs have pro-leukemic effects

**Fig. 4:**
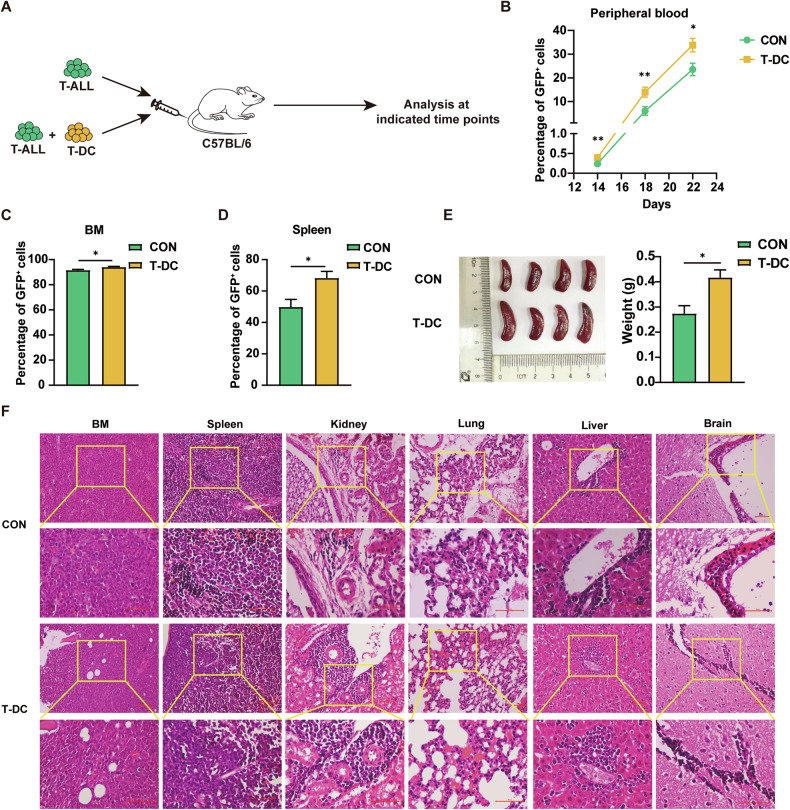
T-DCs have pro-leukemic effects. T-ALL cells were transplanted to recipient mice without (CON) or with sorted T-DCs and the disease progression was monitored. **A** The experimental design is shown. **B** The dynamic distribution of leukemia cells in peripheral blood is shown (*n* = 4). **C**–**F** The leukemia mice were sacrificed on day 18 and the percentage of leukemia cells in the BM and spleen is shown (**C**). The size (**D**) and weight (**E**) of the spleens are shown (*n* = 4). The HE-stained sections of BM, spleen, kidney, lung, liver and brain were detected under a light microscope (**F**). Scale bar, 100 μm. *p < *0.05*, **p < *0.01*.

To study their role on the progression of leukemia and the possible mechanisms, T-DCs were transplanted with leukemia cells (Fig. 4A). Although there was no significant difference in survival time between the two groups (Supplementary Fig. S2A), the leukemia cell levels in PB, BM and spleen of mice transplanted with T-DCs and leukemia cells were significantly higher than the control mice since day 14 after transplantation (Fig. 4B, C). More severe splenomegaly was detected in the T-DCs group than the control group on day 18 (Fig. 4D, E). Pathologic analysis further demonstrated that more infiltrating leukemia cells were detected in the spleen, liver and kidney in the T-DCs group (Fig. 4F). These results demonstrate that T-DCs promote the progression of leukemia.

### Gene expression profile of T-DCs

**Fig. 5:**
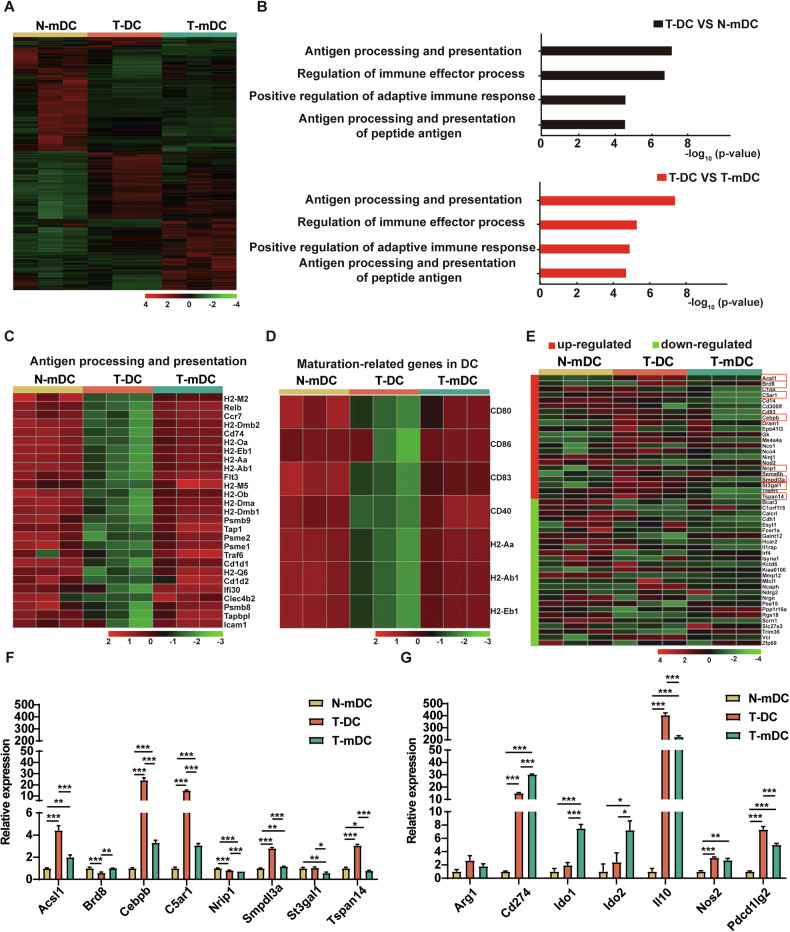
Gene expression profile of T-DCs. N-mDCs, T-DCs and T-mDCs subpopulations were sorted from normal or leukemia mice and RNA-seq analysis was performed. **A** Heat map shows gene expression profiles of N-mDCs, T-DCs and T-mDCs subpopulations (*n* = 3). **B** DEGs of T-DCs vs. N-mDCs and T-DCs vs. T-mDCs were analyzed by GO enrichment. **C** Heat map shows the expression levels of genes from the annotation “Antigen processing and presentation” in N-mDCs, T-DCs and T-mDCs subpopulations. **D** Heat map shows the expression levels of genes from DC maturation-associated genes in N-mDCs, T-DCs and T-mDCs. **E** Heat map shows the expression levels of regulatory DCs signature genes in N-mDCs, T-DCs and T-mDCs. **F**, **G** The expression of selected genes from various database was verified by qRT-PCR. Data are presented as mean ± S.E.M. Unpaired Student’s *t* test, one-way ANOVA tests were used. *p < *0.05*, **p < 0.01, ***p < *0.001*.

To further investigate the characteristics of T-DCs and their mechanisms in leukemia progression, RNA-seq was carried out for gene expression analysis. Heat map exhibited significant differences among T-DCs, N-mDCs and T-mDCs (Fig. 5A). Genome-wide GO analysis showed that DEGs in T-DCs versus N-mDCs or T-mDCs were enriched in annotations “antigen processing and presentation”, “regulation of immune effector process”, “positive regulation of adaptive immune response” and “antigen processing and presentation peptide antigen” (Fig. 5B). Specifically, antigen processing and presentation associated genes and maturation-related genes were selected and presented in heat maps, showing that T-DCs expressed significantly lower levels of those genes than N-mDCs or T-mDCs (Fig. 5C, D). Regulatory DC (DCreg) has been proposed in the literature. We analyzed the expression pattern of a set of DCreg signature genes proposed by Robertson et al. [44] among T-DCs, N-mDCs and T-mDCs. T-DCs show similar expression pattern with DCregs in many genes (Fig. 5E). Different signature genes of DCregs were also suggested [45–47]. However, the analysis showed that T-DCs exhibited opposite expression pattern with DCregs in many genes (Supplementary Fig S3A). The up-regulated genes in Fig. 5E were further verified by qRT-PCR (Fig. 5F). The characteristic genes in Supplementary Fig S3A (Arg1, Cd274, Ido1, Ido2, Il-10, Nos2 and Pdcd1lg2) were also validated by qRT-PCR. It’s worth noting that T-DCs expressed much higher level of Il-10, the well-documented immunosuppressive molecule (Fig. 5G). Taken together, these results demonstrate that T-DCs have similar gene expression pattern with DCregs and might play immunosuppressive roles in T-ALL.

### T-DCs have attenuated phagocytotic and antigen-presentation potential

**Fig. 6:**
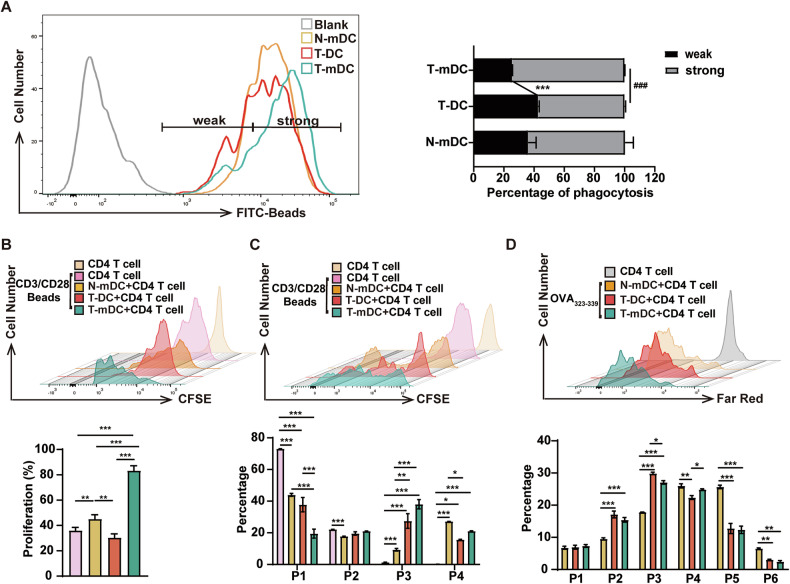
T-DCs have attenuated phagocytotic and antigen-presentation potential. **A** The in vitro phagocytic potential of T-DCs was assessed using latex beads (FITC) uptake experiments. The representative results (left) are shown and the positive rates of weak positive and strong positive (right) are plotted. CFSE-labeled CD4^+^CD44^-^CD62L^+^naïve T cells from either C57BL/6J mice (**B**) or BALB/C (**C**) were co-cultured with N-mDCs, T-DCs and T-mDCs for 5 days. Cell proliferation was assessed by FACS. The representative results (upper) and the percentage of different passages of cells (low) are shown. **D** N-mDCs, T-DCs and T-mDCs were pre-treated with OVA_323-339_ for 1 day and then co-cultured with Far Red-labeled purified OT-II T cells for 5 days. Cell proliferation was assessed by FACS. The representative results (upper) and the percentage of different passages of cells (low) are shown.

Phagocytosis and antigen presentation are two important functions of DCs. As T-DCs express lower levels of antigen presentation and immunomodulation-related genes, we further assessed whether T-DCs were functionally affected. To evaluate the phagocytic function, latex beads uptake experiments were performed. T-DCs had comparable phagocytic potential as N-mDCs. By contrast, T-DCs had lower phagocytic potential than T-mDCs since fewer strong positive cells while more weak-positive cells (gated by fluorescence intensity) were detected in T-DCs than T-mDCs (Fig. 6A). The in vitro DC-naïve T cell co-culture systems were used to test the effects of T-DCs on T cell proliferation. First, DCs were co-cultured with autologous naïve CD4^+^ T cells. T-DCs had lower potential to activate T cells than N-mDCs or T-mDCs (Fig. 6B). Second, allogeneic MLR assays demonstrated that T-DCs were less potent to activate T cells than N-mDCs or T-mDCs, since T cells co-cultured with T-DCs had larger proportion of low-proliferating cells (1-3 generations) and smaller proportion of high-proliferating cells ( > 3 generations) (Fig. 6C). Third, a DC-mediated antigen-specific T-cell proliferation assay was conducted to explore their potential of antigen presentation. Compared with N-mDCs or T-mDCs, T-DCs had attenuated ability to promote specific antigen presentation (Fig. 6D). Therefore, T-DCs are less potent to present antigens and activate T cells than N-mDCs or T-mDCs, which are functionally consistent with immature DCs.

### Single-cell analysis reveals the heterogeneity of T-DCs

**Fig. 7:**
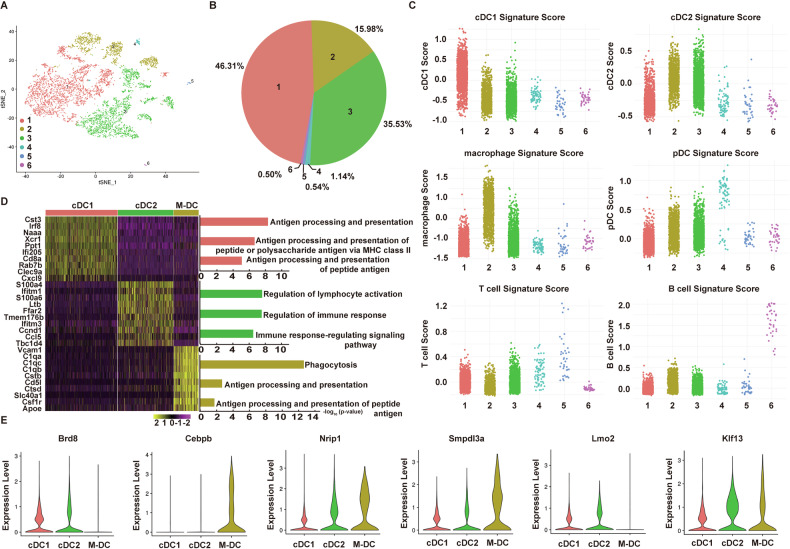
Single-cell analysis reveals the heterogeneity of T-DCs. **A** A total of 7386 successfully profiled T-DC single cells were proceeded *t*-SNE analysis. **B** The distribution of each cluster in T-DCs is shown. **C** The transcriptomic signature of six cell clusters was scored by CMap analysis using specific signatures of cDC1, cDC2, macrophage, pDC, T cell, and B cell. **D** Heat map shows the expression of top 10 cluster-specific genes of each cluster in cDC1, cDC2 and M-DC (left). GO analysis of cluster cDC1, cDC2 and M-DC is shown (right). **E** Violin plots show the expression of DCreg-associated genes in three major clusters in T-DCs.

To further explore the cellular composition and characteristics of T-DCs, scRNA-seq was performed using 10× Genomics technology. A total of 7386 T-DCs passed all quality control filters and were initially analyzed using Cell Ranger. Principal component analysis (PCA) and t-distributed stochastic neighbor embedding (t-SNE) revealed significant transcriptional heterogeneity within the T-DCs population and 6 clusters were identified (Fig. 7A). The pie chart showed that over 97% T-DCs belonged to the clusters 1, 2 and 3, which accounted for 46.31%, 15.98% and 35.53%, respectively (Fig. 7B). To define each cluster, the transcriptomic signature of six cell clusters was scored by Connectivity Map (CMap) analysis using specific signatures of cDC1, cDC2, macrophage, pDC, T cell, and B cell. We compared the transcriptomic signatures of 6 subsets with the CMap scores (scaled dimensionless values) that reflect the extent of similarity between a cell subset and a defined signature gene set. Cluster 1 with the highest score for cDC1 was defined as cDC1, while Cluster 3 with the highest score for cDC2 was defined as cDC2. Cluster 2 had high scores for both cDC2 and macrophage and was classified as macrophage-like DCs (M-DCs). The other three clusters were categorized as pDC, T cell and B cell, respectively (Fig. 7C). The heatmap shows the expression levels of the cluster-specific genes (top 10 genes from each cluster) among the three major cell clusters (Fig. 7D). Meanwhile, the gene expression profiles of three major cell clusters exhibit significantly distinct characteristics. The genes of cDC1 cluster were more enriched in terms related to antigen processing and presentation. Those of cDC2 cluster were more enriched in terms related to regulation of immune response. Those of M-DCs cluster were mainly associated with phagocytosis. As we previously demonstrated that T-DCs had immunosuppressive properties, we further analyzed the expression levels of DCreg-associated immunosuppressive molecules among the three main clusters. The expression levels of Brd8 and Lmo2 were higher in cDC1 or cDC2 than M-DC, whereas the expression levels of Cebpb, Nrip1 and Smpdl3a were higher in M-DC than cDC1 or cDC2 (Fig. 7E). Together, these results demonstrate that T-DCs are heterogeneous population and different cell clusters might exert diverse pathologic roles during leukemia progression.

## Discussion

DCs are involved in both innate and adaptive immunity. They exert anti-tumor activity primarily by antigen presenting whereas participate in the formation of tumor immunomicroenvironment. The maturation of DCs is essential for delivering co-stimulatory signals to T cells and their functions are closely related to their state of maturation [48]. Although DCs also undergo maturation within tumors, it is frequently inadequate for them to elicit a robust immune response, especially under the tumor-associated immunosuppressive conditions [49]. Spleen is an important site residing leukemia cells and a specific leukemia microenvironment is formed during leukemia progression. How leukemia microenvironment imposes DCs and whether leukemia microenvironment educated DCs affect leukemia progression remain insufficiently explored. Here, we studied the characteristics of DCs in the leukemic microenvironment and explored the pathologic roles of those DCs and the mechanisms using a mouse T-ALL model.

Different subtypes of DCs, including imDCs and DCregs, have been identified in the tumor microenvironment playing adverse roles [50]. The accumulation of imDCs, defined by both phenotypic and functional characteristics, in tumors is often driven by factors in the microenvironment, such as hypoxia and lactate, which skew DC maturation pathways. ImDCs express low levels of MHCII molecules and co-stimulatory molecules such as CD80 (B7.1), CD86 (B7.2) and CD83 (HB15) on their surface [14]. Furthermore, tumor microenvironment induces imDCs to secret suppressive factors like Il-10, Arg1, Ido1 and Ido2 [51]. Here, we identified a population of CD11c^+^MHCII^lo^ cells (T-DCs) accumulated in the splenic microenvironment when we analyzed the characteristics of DCs in a mouse T-ALL model. T-DCs exhibited an immature phenotype as they expressed low levels of CD86, CD83, CD40 and MHCII. These results implied that T-ALL microenvironment hindered DC maturation. In addition, they also expressed high levels of immunosuppressive molecules like Il-10 and PD-1. Therefore, we preliminarily classified T-DCs as imDC. Nevertheless, DCregs with immunosuppressive characteristics have also been identified in tumor microenvironment. DCregs are characterized by low level expression of CD86, CD40 and CD83, existence at various maturation stages, and exerting immune suppressive roles through diverse mechanisms. Interestingly, T-DCs share some similar features with DCregs, including similar expression patterns of some DCreg signature genes and impaired antigen presentation to CD4^+^ T cells. Importantly, T-DCs accelerated leukemia progression in vivo. Therefore, T-DCs are imDCs sharing immunosuppressive characteristics with DCregs in the leukemia microenvironment. It was reported that blocking immunosuppressive molecules could restore the antigen-presenting function of DCregs, and enable them to effectively activate T cells [52]. Further research is warranted to explore the mechanisms by which leukemia microenvironment hinders DC maturation and to develop targeted therapies that restore the maturation of DCs as well as decrease the expression of immunosuppressive molecules for better clinical treatment in leukemia.

High level of imDCs correlates with hindered immune response and adverse clinical outcome in solid tumors [7, 9]. Given the absence of subset-specific data on DCs in human databases, a DC maturation-associated gene set defined in the literature was used for the survival analysis on an open clinical database [14, 53, 54]. Patients with lower score of this gene set had worse survival. Furthermore, the analysis for single gene in the gene set revealed that low level expression of CD83, HLA-DMB, HLA-DQB1, HLA-DRA or HLA-DRB1, which was also detected in T-DCs (CD83 and MHCII), was significantly correlated with poor prognosis in ALL patients. Although neither the maturation-associated gene score nor the level of any above-mentioned single gene definitively reflects the level of either T-DCs or imDCs, it gives a possible clue that high level of T-DCs and imDCs might be an adverse factor in disease progression. Our findings are in line with recent studies that highlight the functional interaction between AML and the immune system, where immune evasion by leukemia cells is a significant factor in disease progression [55]. This suggests that the maturation status of DCs may have significant implications for the efficacy of immune responses against leukemia. Likewise, in vitro treatment of T-DCs with maturation inducers, GM-CSF alone or in combination with TNF-α and IL-1β, promoted their maturation, as shown by increased MHCII^+^ cells and upregulated expressions of CD80, CD86, CD83, and CD40. While these inducers may enhance DC function, whether they can effectively alter disease progression or contribute to improved outcomes in leukemia models has yet to be fully established. Further investigation should cover how leukemia microenvironment hinders DC maturation and how to develop strategies restoring the maturation of DCs.

DCs are composed of cDCs and pDCs [56]. Furthermore, in tumor microenvironment, DCs exhibit great heterogeneity in multifaceted aspects including different stages of maturation, and functional specialization, *etc*. T-DCs possess the characteristics of both imDCs and DCregs, both of which have immunosuppressive function. However, whether T-DCs are still a heterogeneous population has not yet been characterized. The scRNA-seq can resolve inter-subgroup heterogeneity and provide gene expression profiles at the single-cell level. Here, we identified three main subpopulations, *i.e*. cDC1, cDC2 and M-DCs as well as three tiny subpopulations, *i.e*. pDCs, T cells and B cells. The M-DCs were first described in the in vitro DC-induction experiments when myeloid BM precursors from NOD and NOR mice were induced by GM-CSF. A CD11c^+^MHCII^low^ imDC population was identified and suggested as an M-DC subset [57]. In fact, cDCs and macrophages have close relationship as they share same ontogeny and most differentiation pathways, and have considerable functional similarities [58]. T-DCs had antigen cross-presentation capabilities, which is the most important characteristics of DCs. Therefore, although this subpopulation of T-DCs share transcriptional similarities with both cDC2 and macrophages, we prefer defining them as M-DCs. Notably, both cDC1 and cDC2 subsets were found to predominantly express immune-suppressive molecules whereas the M-DC subset had phagocytotic characteristics. M-DCs exhibit characteristics of both macrophages and DCs, suggesting a functional plasticity that is particularly relevant in the dynamic leukemia microenvironment. Further research on the specific roles of each subset and interactions with leukemia cells and other immune components could provide valuable insights for the exploration of effective immunotherapeutic strategies.

Taken together, we identified a new subpopulation of DCs, CD11c^+^MHCII^lo^ DCs, accumulated in the microenvironment in a mouse T-ALL model. T-DCs exhibit an immature phenotype as they expressed low levels of CD83, CD86, MHCII and CD40. More immature DCs correlated with poor prognosis in leukemia patients. Functional analysis revealed that T-DCs had pro-leukemia potential. They promoted T-ALL progression by inhibiting CD4⁺ T cell activation and expressing high levels of immunosuppressive molecules. Our findings demonstrate that the leukemia microenvironment significantly influences DC maturation, and in turn, these imDCs actively contribute to leukemia progression. Our finding has significant implications for the development of immunotherapeutic strategies targeting the leukemic microenvironment.

## Supplementary information

Supplementary figure1

Supplementary figure2

Supplementary figure3

Supplementary table1

Supplementary table2

Supplementary table3

Supplementary table4

## Data Availability

All datasets in the present study can be obtained from the corresponding author upon request.
