# Correction: Identification of a novel immature dendritic cell subset with potential pro-leukemic effects in leukemia microenvironment

**DOI:** 10.1038/s41419-025-08137-3

**Published:** 2025-11-03

**Authors:** Xiaoxi Cui, Yifei Li, Wanzhen Xie, Ruiyun Li, Dongyue Zhang, Siqi Zhang, Qian Ren, Lina Wang, Guoguang Zheng

**Affiliations:** 1 State Key Laboratory of Experimental Hematology, National Clinical Research Center for Blood Diseases, Haihe Laboratory of Cell Ecosystem, Institute of Hematology & Blood Diseases Hospital, Chinese Academy of Medical Sciences & Peking Union Medical College, Tianjin, China; 2 Tianjin Institutes of Health Science, Tianjin, China

**Keywords:** Cancer microenvironment, Dendritic cells

Correction to: *Cell Death & Disease* 10.1038/s41419-025-07851-2, published online 29 July 2025

In this article, the caption of figure 6 should read:

CFSE-labeled CD4^+^CD44^-^CD62L^+^naïve T cells from either C57BL/6J mice.

Instead of:

CFSE-labeled CD4+CD44+CD62L- CD44 - CD62L+naïve T cells from either C57BL/6J mice.

The original article has been updated.

