# Supplementary material for: Identification of a novel immature dendritic cell subset with potential pro-leukemic effects in leukemia microenvironment
Source: Cell Death Dis. 2025 Jul 29;16(1):571. doi: 10.1038/s41419-025-07851-2 (PMC12307975; doi:10.1038/s41419-025-07851-2)
Supplement: Supplementary file 1 — Supplementary figure1 [file 41419_2025_7851_MOESM1_ESM.docx]

**Supplementary Figure 1**


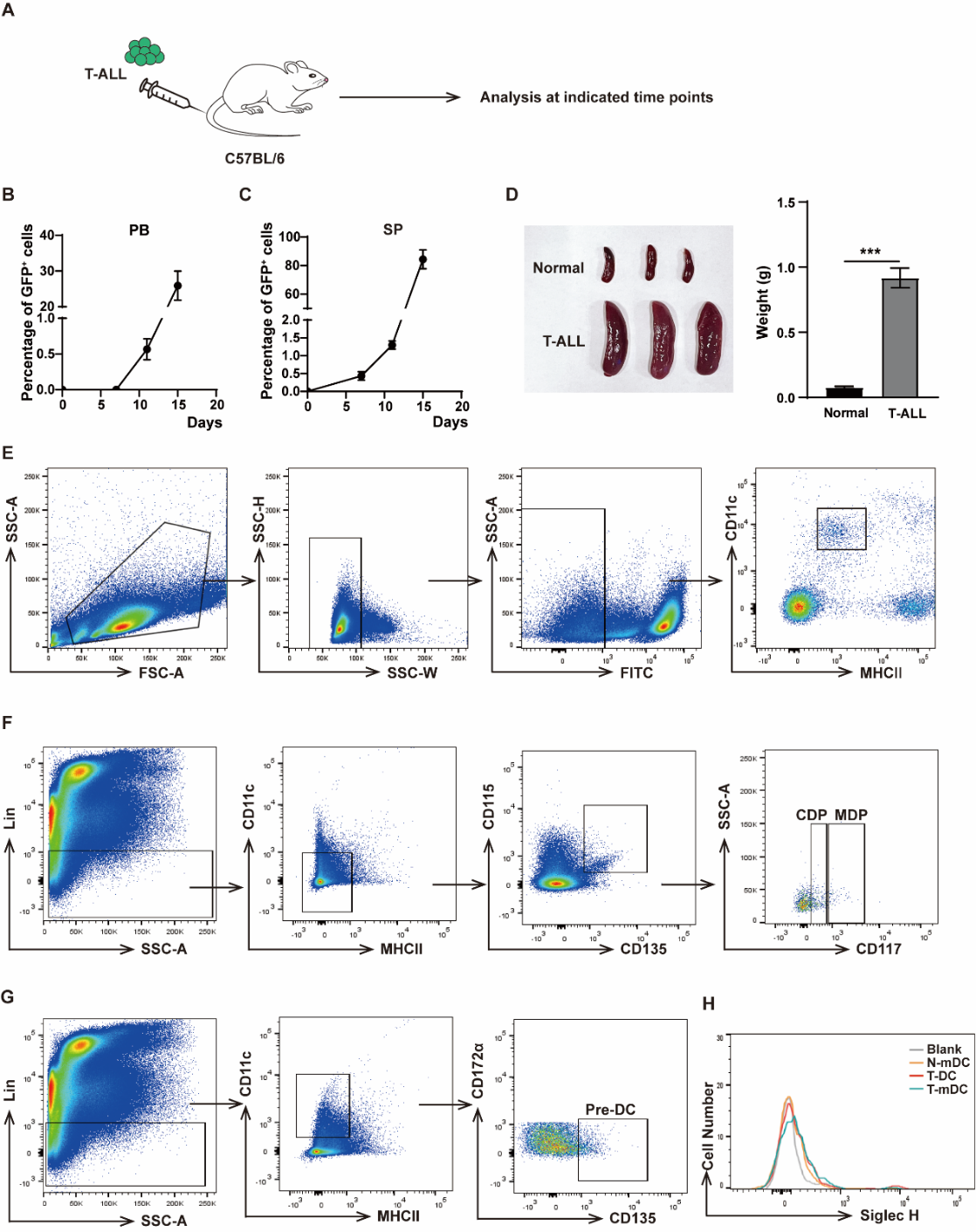


**Fig. S1 Establishment of mouse T-ALL model and gating strategy for cDCs**

**A.** The experimental design of the establishment of mouse T-ALL model. **B-C.** The percentage of PB (B) and spleen (C) GFP^+^ leukemia cells in recipient mice at indicated time points was detected by flow cytometry (n=5). **D.** The size (left) and weight (right) of spleens from normal and T-ALL mice are shown. **E**. The gating strategy for sorting T-DCs from the GFP^-^CD3^-^CD19^-^NK1.1^-^ population by flow cytometry. **F-G.** The gating strategy for sorting MDPs, CDPs (F) and Pre-DCs (G) by flow cytometry.  **H.** The expression of Siglec H in N-mDCs, T-DCs and T-mDCs was measured by flow cytometric. The data are representative of three independent experiments. ****p<0.001.*
