# Supplementary material for: Identification of a novel immature dendritic cell subset with potential pro-leukemic effects in leukemia microenvironment
Source: Cell Death Dis. 2025 Jul 29;16(1):571. doi: 10.1038/s41419-025-07851-2 (PMC12307975; doi:10.1038/s41419-025-07851-2)
Supplement: Supplementary file 2 — Supplementary figure2 [file 41419_2025_7851_MOESM2_ESM.docx]

**Supplementary Figure 2**


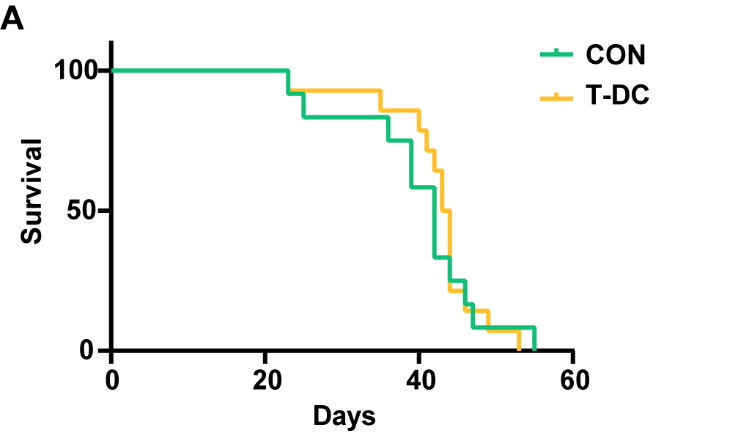


**Fig. S2 The impact of T-DCs on the survival of leukemia mice**

T-ALL cells were transplanted to recipient mice without (CON) or with sorted T-DCs and the survival of mice were compared by Kaplan–Meier curves (n=10 for each group). Kaplan–Meier estimates were used.
