# Supplementary material for: Identification of a novel immature dendritic cell subset with potential pro-leukemic effects in leukemia microenvironment
Source: Cell Death Dis. 2025 Jul 29;16(1):571. doi: 10.1038/s41419-025-07851-2 (PMC12307975; doi:10.1038/s41419-025-07851-2)
Supplement: Supplementary file 3 — Supplementary figure3 [file 41419_2025_7851_MOESM3_ESM.docx]

**Supplementary Figure 3**


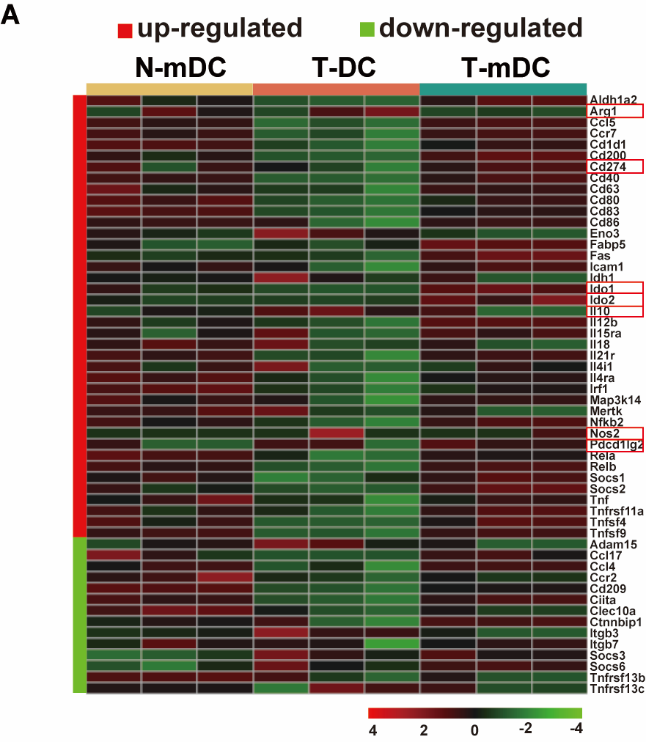


**Fig. S3 Gene expression profile of T-DCs**

Heat map shows the expression levels of regulatory DCs signature genes in N-mDCs, T-DCs and T-mDCs.
