# Supplementary material for: Identification of a novel immature dendritic cell subset with potential pro-leukemic effects in leukemia microenvironment
Source: Cell Death Dis. 2025 Jul 29;16(1):571. doi: 10.1038/s41419-025-07851-2 (PMC12307975; doi:10.1038/s41419-025-07851-2)
Supplement: Supplementary file 4 — Supplementary table1 [file 41419_2025_7851_MOESM4_ESM.docx]

| **Antibody** | **Clone** | **Fluorescence** | **Manufacture** |
| --- | --- | --- | --- |
| I-a^b^ | AF6-120 | PE  APC-CY7 | BioLegend |
| CD11c | N418 | APC  APC-CY7 | BioLegend |
| CD11b | M1/70 | PerCP-Cy5.5 | BioLegend |
| F4/80 | BM8 | PerCP-Cy5.5 | eBioscience |
| CD172α | P84 | APC-CY7 | BioLegend |
| CD24 | M1-69 | PE-CY7 | BD Bioscience |
| CD115 | AFS98 | PE-CY7 | BioLegend |
| CD40  CD83  CD80  CD86 | 3-23  Michel-19  16-10A1  GL-1 | Pacific Blue  PE-CY7  BV421  APC-CY7 | BioLegend  BioLegend  BioLegend  BioLegend |
| CD45.1 | A20 | APC-CY7 | BioLegend |
| CD45.2 | 104 | PerCP-Cy5.5 | BioLegend |
| SiglecH | eBio440c | APC | BioLegend |
| CD4  CD3  CD19  NK1.1 | GK1.5  17A2  1D3  PK136 | APC  FITC  FITC  FITC | BioLegend  BioLegend  BioLegend  BioLegend |
| CD44 | IM7 | PE | BioLegend |
| 7-AAD |  | PerCP-Cy5.5 | BD Bioscience |
| B220  CD62L  PD-1  CD135  CD117  Ly6G | RA3-6B2  MEL-14  J43  A2F10  2B8  1A8-Ly6g | PE  V450  PE-CY7  PE  PE-CY7  FITC | BioLegend  BD Bioscience  BD Bioscience  BioLegend  BioLegend  eBioscience |

**Supplementary Table S1. Antibodies used in flow cytometry**
