# Supplementary material for: Identification of a novel immature dendritic cell subset with potential pro-leukemic effects in leukemia microenvironment
Source: Cell Death Dis. 2025 Jul 29;16(1):571. doi: 10.1038/s41419-025-07851-2 (PMC12307975; doi:10.1038/s41419-025-07851-2)
Supplement: Supplementary file 5 — Supplementary table2 [file 41419_2025_7851_MOESM5_ESM.docx]

**Supplementary Table S2. Primer sequence**

| **Gene** | **Forward (5’-3’)** | **Reverse (3’-5’)** |
| --- | --- | --- |
| **Mouse genes** |  |  |
| Arg1 | GATTATCGGAGCGCCTTTCT | CCACACTGACTCTTCCATTCTT |
| Acsl1 | TGGGGTGGAAATCATCAGCC | GGGAACCACAGGGAAGATGG |
| Brd8 | CAGCAATTCTTGGCCACAC | ATCTGCTTCAATGGCACAGC |
| Cd274 | CTACGGTGGTGCGGACTACA | CAGACTGCTGGTCACATTGAGAAG |
| Cebpb | GACAAGCTGAGCGACGAGTA | TGCTTGAACAAGTTCCGCAG |
| C5ar1 | AGGTCTCTCCCCAGCATCAT | GTCGTGGACGGAGTGAAAGT |
| Ido1 | ATGTGGGCTTTGCTCTACCA | AAGCTGCCCGTTCTCAATCA |
| Ido2 | ACTCTGACCTGGTGCTGACAA | CCTGACTGTGTTGCCGAATGG |
| Il-10 | GGCCCAGAAATCAAGGAGCA | GCCTTGTAGACACCTTGGTCTT |
| Nos2 | TCACCTTCGAGGGCAGCCGA | TCCGTGGCAAAGCGAGCCAG |
| Pdcd1lg2 | CCCTAAAGAAGTGTACACCGTA | CTTCTGCAAACTGGCTCTTATC |
| Smpdl3 | CTTTGCTGCCTACTGGTTGC | GGAGGCATTTGCGCCTTTAG |
| St3gal1 | CTGACAGTCCACAACGCTCT | GCTGCCAGGGTAGTTGTGAT |
| Gapdh | ACTCCACTCACGGCAAATTCAAC | GACACCAGTAGACTCCACGACAT |
