# Supplementary material for: Identification of a novel immature dendritic cell subset with potential pro-leukemic effects in leukemia microenvironment
Source: Cell Death Dis. 2025 Jul 29;16(1):571. doi: 10.1038/s41419-025-07851-2 (PMC12307975; doi:10.1038/s41419-025-07851-2)
Supplement: Supplementary file 6 — Supplementary table3 [file 41419_2025_7851_MOESM6_ESM.docx]

**Supplementary Table S3. DC maturation-associated gene set**

| **Number** | **Gene** |
| --- | --- |
| 1  2  3  4  5  6  7 | CCL19  CCL21  CCR7  CD209  CD40  CD80  CD83 |
| 8  9 | CD86  HLA-DMB |
| 10  11 | HLA-DRA1  HLA-DRB1 |
| 12 | HLA-DQB1 |
| 13 | RELB |
