# Supplementary material for: Identification of a novel immature dendritic cell subset with potential pro-leukemic effects in leukemia microenvironment
Source: Cell Death Dis. 2025 Jul 29;16(1):571. doi: 10.1038/s41419-025-07851-2 (PMC12307975; doi:10.1038/s41419-025-07851-2)
Supplement: Supplementary file 7 — Supplementary table4 [file 41419_2025_7851_MOESM7_ESM.docx]

**Supplementary Table S4. Cell signature genes**

| **Signature type** | **Gene** |
| --- | --- |
| cDC1 Signature genes | Batf3, Cadm1, Clec9a, H2-k1, Irf4, Irf8, Itgax, Lrrc3, Tap1, Tlr3, Wdfy4, Xcr1 |
| cDC2 Signature genes | Aif1, Cd11b, Cd301b, Clec10a, Cx3cr1, Fcgr2b, Il12b, Irf4, Lst1, Pdcd1lg2, Sirpa, S100a4, Tlr4, Ywhah |
| Macrophage Signature genes | Adgre1, Apoe, C1qa, C1qb, Ccr2, Cd163, Cd68, Csf1r, Cx3cr1, Il10, Marco, Mertk, Tnf |
| pDC Signature genes | Bst2, Clec4c, Gzmb, Ifna, Il3ra, Irf7, Lilrb4a, Nrp1, Ptprc, Siglech, Spib, Tlr7, Tlr9 |
| T cell Signature genes | Cd3e, Cd4, Cd8a, Foxp3, Gzmb, Ifng, Il4, Il17a, Klrd1, Nkg7, Pdcd1, Tnf |
| B cell Signature genes | Bank1, Cd19, Cd27, Cd38, Cd79a, Cd79b, Igha, Ighg1, Ighm, Ms4a1, Pax5 |
